# Comment on Patsaki et al. Benefits from Incorporating Virtual Reality in Pulmonary Rehabilitation of COPD Patients: A Systematic Review and Meta-Analysis. *Adv. Respir. Med.* 2023, *91*, 324–336

**DOI:** 10.3390/arm92010002

**Published:** 2023-12-20

**Authors:** Mostafa Salimi

**Affiliations:** Students Research Committee, Faculty of Medicine, Mashhad Medical Sciences, Islamic Azad University, Mashhad 913376351, Iran; salimi.mostafa@iaumshms.ac.ir

I am writing this comment regarding the review article by Patsaki et al. titled “Benefits from Incorporating Virtual Reality in Pulmonary Rehabilitation of COPD Patients: A Systematic Review and Meta-Analysis” [1] that was published on 10 August 2023. I have read the article with great interest and appreciate the authors’ efforts to synthesize the evidence on this topic. However, I have noticed a mistake in the meta-analysis of the 6-minute walk distance (6MWD) variable that may affect the validity of the results and conclusions.

The mistake is related to the data extraction of the study by Sutanto et al. [2], which was one of the included trials in the meta-analysis. The authors reported that they used the mean and standard deviation of the 6MWD for both the control and experimental groups from Sutanto et al. However, when I checked the original paper by Sutanto et al., I found that the authors of the review article had mistakenly entered the control group data in place of the experimental group data, and vice versa, in their statistical software. This means that they had reversed the effect of virtual reality on 6MWD in their meta-analysis. In addition, the sample sizes of both groups appear to have been incorrectly extracted.

To verify this, I re-calculated the pooled mean difference and 95% confidence interval for 6MWD using the correct data from Sutanto et al. and the other included studies. I used Comprehensive Meta-Analysis Version 3 software [3], which I obtained from Biostat, and a random effects model. I found that the corrected pooled mean difference was 95% CI = 15.52 (−1.20 to 32.25), which was not statistically significant (*p* = 0.069). This is different from the reported pooled mean difference (95% CI = 22.7 (19.92 to 25.63)), which was statistically significant (*p* < 0.001).

This mistake has important implications for the interpretation and application of the review article. The authors concluded that virtual reality had a positive effect on 6MWD in COPD patients, which is a clinically relevant outcome measure for pulmonary rehabilitation. However, based on my re-analysis, this conclusion is not supported by the data and should be revised. Furthermore, this mistake may also affect the results of other outcomes that were meta-analyzed using data from Sutanto et al., including quality of life and dyspnea. According to Patsaki et al.'s meta-analysis, virtual reality had a significant negative effect on dyspnea; the pooled mean difference was 95% CI = −0.06 (−0.36 to 0.24), *p* = 0.07.

I also wonder how this mistake occurred, as data extraction should be performed by at least two authors independently and any discrepancies should be resolved by consensus or a third author. This raises some concerns about the quality and rigor of the systematic review method used by the authors of the review article.

I request that a correction or an erratum be published to address this mistake and inform the readers about it. I also suggest that the authors of the review article check their data extraction and analysis for other studies to ensure their accuracy and validity.
